# Comparative Functional Analysis of Class II Potassium Transporters, SvHKT2;1, SvHKT2;2, and HvHKT2;1, on Ionic Transport and Salt Tolerance in Transgenic Arabidopsis

**DOI:** 10.3390/plants9060786

**Published:** 2020-06-23

**Authors:** Yuichi Tada, Aki Ohnuma

**Affiliations:** School of Bioscience and Biotechnology, Tokyo University of Technology, 1404-1 Katakura, Hachioji, Tokyo 192-0982, Japan; b011503974@edu.teu.ac.jp

**Keywords:** Arabidopsis, barley, high-affinity potassium transporter (HKT), salt tolerance, *Sporobolus virginicus*

## Abstract

Class II high-affinity potassium transporters (HKT2s) mediate Na^+^–K^+^ cotransport and Na^+^/K^+^ homeostasis under K^+^-starved or saline conditions. Their functions have been studied in yeast and *X. laevis* oocytes; however, little is known about their respective properties in plant cells. In this study, we characterized the Na^+^ and K^+^ transport properties of SvHKT2;1, SvHKT2;2 and HvHKT2;1 in Arabidopsis under different ionic conditions. The differences were detected in shoot K^+^ accumulation and root K^+^ uptake under salt stress conditions, K^+^ accumulation in roots and phloem sap under K^+^-starved conditions, and shoot and root Na^+^ accumulation under K^+^-starved conditions among the *HKT2s* transgenic lines and WT plants. These results indicate the diverse ionic transport properties of these HKT2s in plant cells, which could not be detected using yeast or *X. laevis* oocytes. Furthermore, Arabidopsis expressing *HKT2s* showed reduced salt tolerance, while over-expression of *HvHKT2;1* in barley, which has the ability to sequestrate Na^+^, showed enhanced salt tolerance by accumulating Na^+^ in the shoots. These results suggest that the coordinated enhancement of Na^+^ accumulation and sequestration mechanisms in shoots could be a promising strategy to confer salt tolerance to glycophytes.

## 1. Introduction

Soil salinity is a severe environmental stress factor, causing significant losses in global agricultural productivity, particularly in irrigated soils [1,2,3]. Plant growth under salt stress requires rigorous control of the uptake, long-distance transport, and accumulation of K^+^ and Na^+^ ions. When combined, the transport systems involved in the uptake and distribution of K^+^ and Na^+^ act as key determinants of plant salt tolerance due to their ability to determine tissue and cytosolic K^+^/Na^+^ ratios, parameters that are generally believed to impact greatly on salt tolerance [4,5]. In general, Na^+^ is excluded from the shoots and K^+^ accumulates in glycophytes under salinity stress, thereby maintaining the high cytosolic K^+^/Na^+^ ratio, particularly in leaves [6,7,8,9,10]. However, halophytes can select K^+^ from a Na^+^-dominated mixture, but also accumulate enough Na^+^ to enable osmotic adjustment [11]. Barley *(Hordeum vulgare*) has been described as a salt includer, and is sometimes considered a halophyte [12,13]. Salt-induced K^+^ efflux in barley mesophyll cells in salt-sensitive varieties was higher than in salt-tolerant varieties [14,15], and in contrast to cultivated barley, halophytic wild barley relatives exhibited significantly higher tissue K^+^ retention [16].

HKTs permeable either to Na^+^ only (class I) or K^+^ and Na^+^ (class II) are thought to play a major role in the uptake and distribution of K^+^ and Na^+^ in plant tissues. Class I HKTs play a role in Na^+^ excretion from the xylem sap and recirculation from the leaves to the roots via the phloem vasculature [6,7,9,17,18,19]. Class II HKTs are thought to mediate Na^+^–K^+^ cotransport in plants and Na^+^–K^+^ homeostasis under K^+^-starved and saline conditions. These HKTs are predominantly found in monocot species [13,20,21,22,23,24,25,26]. Each class II HKT demonstrated a distinct expression profile and ion selectivity under different K^+^ and Na^+^ concentrations. TaHKT2;1 from wheat is permeable to both Na^+^ and K^+^ [23,27] and expressed in the root cortical cells [24]. A decrease in *TaHKT2;1* expression levels in transgenic wheat resulted in lower root Na^+^ uptake and reduced translocation into xylem sap, leading to higher salt tolerance [28]. OsHKT2;1 mediated Na^+^ uptake, and OsHKT2;2 mediated Na^+^-K^+^ cotransport in cultured tobacco cells [29]. Arabidopsis overexpressing *PutHKT2;1* from *Puccinellia tenuiflora* or *OsHKT2;1* showed increased sensitivities to Na^+^ and PutHKT2;1 or OsHKT2;1 were involved in Na^+^-uptake at higher external K^+^ concentrations or low external K^+^ concentrations, respectively [20]. The overexpression of *HvHKT2;1* in barley also leads to higher Na^+^ concentrations in xylem sap under normal conditions and higher K^+^ concentration in the leaf blade under 50 mM Na^+^ [13]. We previously reported that the halophytic turf grass *Sporobolus virginicus* demonstrated remarkable salt tolerance (up to 1.5 M NaCl) and maintained a high K^+^/Na^+^ ratio even under salt stress [30]. We found that SvHKT2;1 and SvHKT2;2 (SvHKT2s) from this halophyte complemented K^+^-uptake deficiency in mutant yeast and mediated K^+^ and Na^+^ transport to both inward and outward in *Xenopus* *laevis* oocytes [25]. *Arabidopsis* constitutively expressing SvHKT2s accumulated increased amount of K^+^ and Na^+^ in shoots under K^+^-starved conditions, while K^+^ concentration in the xylem sap of transformants was also higher than in wild type plants. These results suggested the presence of enhanced K^+^ and Na^+^ uploading to xylem from xylem parenchyma cells and functional similarity between HvHKT2;1 and SvHKT2s.

In this study, we performed a comparative functional analysis of potassium transporters, SvHKT2;1, SvHKT2;2 and HvHKT2;1 ectopically expressed in transgenic Arabidopsis plants under different ionic conditions to reveal their functional similarities and differences. We also examined the effects of the ectopic expression of these transporter genes on salt tolerance in transgenic Arabidopsis plants. Analyzing Na^+^ and K^+^ transporting property of halophytic HKT transporters in plant cells may reveal their key functions and could bring novel insights into the strategies for breeding salt-tolerant plants.

## 2. Results

### 2.1. Root Growth under K^+^-Starved Conditions

**Figure 1:**
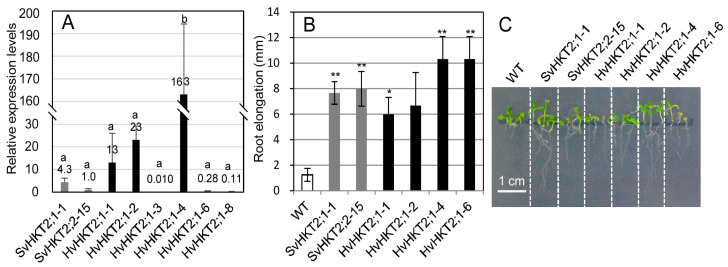
Expression levels of the transgenes and root growth of the transgenic lines. (**A**) Expression levels of transgenes in the SvHKT2;1-1, SvHKT2;2-15, and HvHKT2;1 lines were determined by qRT-PCR. Expression levels are shown relative to that of the *SvHKT2;2* gene in the SvHKT2;2-15 line. Data are presented as means ± SE (n = 3, biological replicates). Means with a different letter are significantly different at *p* < 0.05 using the Tukey’s test. (**B**) Root elongation of transgenic and WT seedlings grown on 0.1 mM K^+^ medium during 5 days from 5 days to 10 days after sowing. Data are presented as means ± SE (n = 3, biological replicates). Single and double asterisks denote significant differences compared to the values from WT plants at *p* < 0.05 and *p* < 0.01, respectively, determined using the Student’s *t*-test. (**C**) The appearance of transgenic lines and WT seedlings at 10 days after sowing on 0.1 mM K^+^ medium.

We previously produced and characterized transgenic *Arabidopsis* lines expressing *35S-SvHKT2;1* and *35S-SvHKT2;2*; we also selected representative lines (SvHKT2;1-1 and SvHKT2;2-15, respectively) and reported that their root growth was promoted compared with WT under K^+^-starved conditions [25]. In this study, we used these two representative lines as typical *SvHKT2* transformants and newly produced transgenic *Arabidopsis* lines expressing *35S-HvHKT2;1.* We compared the expression levels of the transgene in six *HvHKT2;1* lines with those of the *SvHKT2* lines grown on the 1/2 MS agar medium (Figure 1A). No HvHKT2;1 line showed similar levels of transgene expression as SvHKT2;1-1 and SvHKT2;2-15; therefore, we selected three lines (HvHKT2;1-1, HvHKT2;1-2, and HvHKT2;1-4) with higher transgene expression compared to SvHKT2 transformants, and an additional line (HvHKT2;1-6) with lower expression levels than SvHKT2 transformants and used for root growth experiment. When the transgenic lines and WT were grown on a 0.1 mM K^+^ medium, HvHKT2;1 lines, as well as SvHKT2 lines, showed enhanced root growth compared with WT plants, regardless of their transgene expression levels (Figure 1B,C). Thus, the ectopic expression of *SvHKT2s* and *HvHKT2;1* transporters had a similar influence on root growth under K^+^-starved conditions.

### 2.2. Ionic Concentrations in Transgenic Arabidopsis

**Figure 2:**
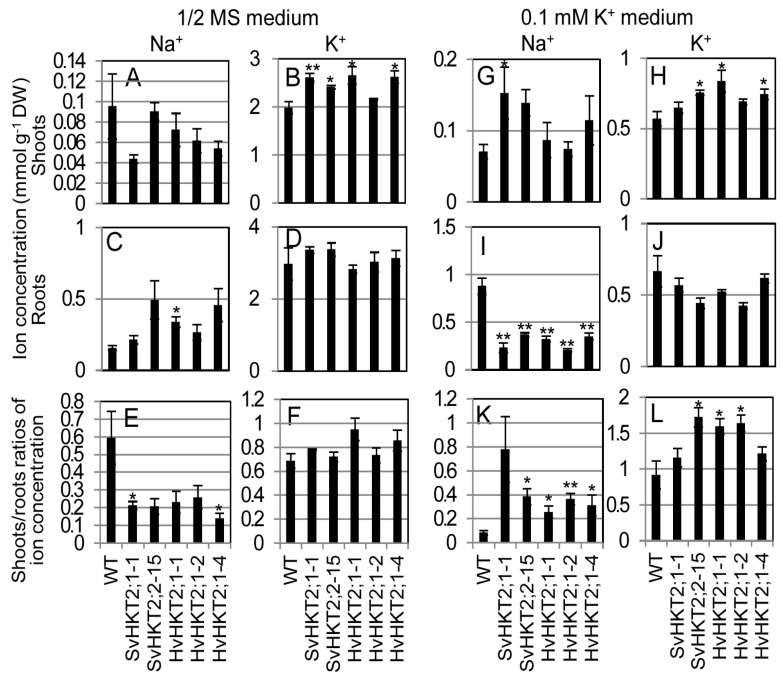
Na^+^ and K^+^ concentrations and shoots/roots ratios of Na^+^ and K^+^ concentrations in the *SvHKT2s* and *HvHKT2;1* transgenic lines and WT plants grown in 1/2 MS or 0.1 mM K^+^ medium. Two-week-old seedlings germinated on 1/2 MS medium were hydroponically cultured in 1/2 MS (**A**–**F**) or 0.1 mM K^+^ (**G**–**L**) medium for an additional two weeks, and the Na^+^ (**A**,**C**,**G**,**I**) and K^+^ (**B**,**D**,**H**,**J**) concentrations in their shoots (**A**,**B**,**G**,**H**) and roots (**C**,**D**,**I**,**J**) and shoots/roots ratios of Na^+^ (**E**,**K**) and K^+^ (**F**,**L**) concentrations were determined. Data are presented as means ± SE (n = 3–4, biological replicates). Single and double asterisks denote significant differences compared with the values of WT plants at *p* < 0.05 and *p* < 0.01, respectively, determined using the Student’s *t*-test

To compare the transporter activity of SvHKT2s and HvHKT2;1 in Arabidopsis plants, we determined ionic concentrations in shoots and roots of *SvHKT2s* and *HvHKT2;1* lines cultured in 1/2 MS (0.2 mM Na^+^ and 10 mM K^+^) or 0.1 mM K^+^ medium (0.725 mM Na^+^) (Figure 2).

In normal conditions (1/2 MS medium), no significant differences were detected in Na^+^ concentrations in either shoots or roots, nor in K^+^ concentrations in roots between the *SvHKT2s* and *HvHKT2;1* lines and WT plants, except for root Na^+^ concentration in HvHKT2;1-1 (Figure 2A,C,D). However, K^+^ concentration in the shoots was higher in the *SvHKT2s* and *HvHKT2;1* lines than in WT plants (Figure 2B). Consequently, shoots/roots Na^+^ ratios in the transgenic lines were significantly lower than in WT plants, but those for K^+^ were not significantly different between the transgenic lines and WT plants (Figure 2E,F). 

In K^+^-starved conditions (0.1 mM K^+^ medium), shoot Na^+^ and K^+^ concentrations in *SvHKT2s* and *HvHKT2;1* lines were significantly higher or tended to be higher than those of WT plants, with *SvHKT2s* lines showing a significantly higher accumulation of shoot Na^+^ (Figure 2G,H). Interestingly, root Na^+^ concentrations in the transgenic lines were significantly lower than those in WT plants (Figure 2I), while no significant differences were observed in K^+^ concentrations between the transgenic lines and WT plants (Figure 2J). Consequently, the shoots/roots ratio of Na^+^ and K^+^ concentrations in both transgenic lines was higher than in WT (Figure 2K,L). These results were consistent with our previous report on ionic concentrations in *SvHKT2* lines under K^+^-starved conditions [25].

### 2.3. Salt Tolerance in Transgenic Arabidopsis

**Figure 3:**
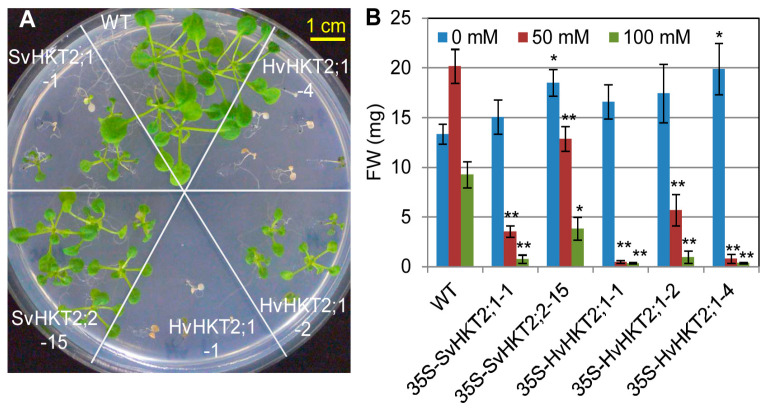
Salt tolerance test for SvHKT2s and HvHKT2;1 transgenic lines. One-week-old seedlings germinated on 1/2 MS agar medium were transplanted onto 1/2 MS agar medium supplemented with 0, 50, or 100 mM NaCl and their fresh weight (FW) was determined after another two weeks of incubation. (**A**) The appearance of transgenic lines and WT plants on 1/2 MS medium supplemented with 100 mM NaCl. (**B**) FW of transgenic lines and WT plants incubated on 1/2 MS medium supplemented with different concentrations of NaCl. Data are presented as means ± SE (n = 6, biological replicates). Single and double asterisks denote significant differences compared with the values of WT plants of the same conditions at *p* < 0.05 and *p* < 0.01, respectively, determined using the Student’s *t*-test.

We evaluated salt tolerance in transgenic Arabidopsis lines constitutively expressing *SvHKT2s* or *HvHKT2;1.* When seedlings of transgenic lines and WT plants were transferred onto an agar medium supplemented with different concentrations of NaCl, all transgenic lines showed higher salt sensitivity (i.e., reduced salt tolerance) compared to that of WT plants (Figure 3). The growth of both *SvHKT2* and *HvHKT2;1* lines was severely inhibited at 50 and 100 mM NaCl compared to those in WT, but no adverse effect was observed on the transgenic lines at 0 mM NaCl (Figure 3B). All *HvHKT2;1* lines tested showed higher transgene expression than *SvHKT2* lines (Figure 1A) and showed high salt-sensitivity or died at 100 mM NaCl (Figure 3A,B). Under the experimental conditions of this work, adding 50 mM NaCl to the culture medium unexpectedly increased FW of WT plants.

**Figure 4:**
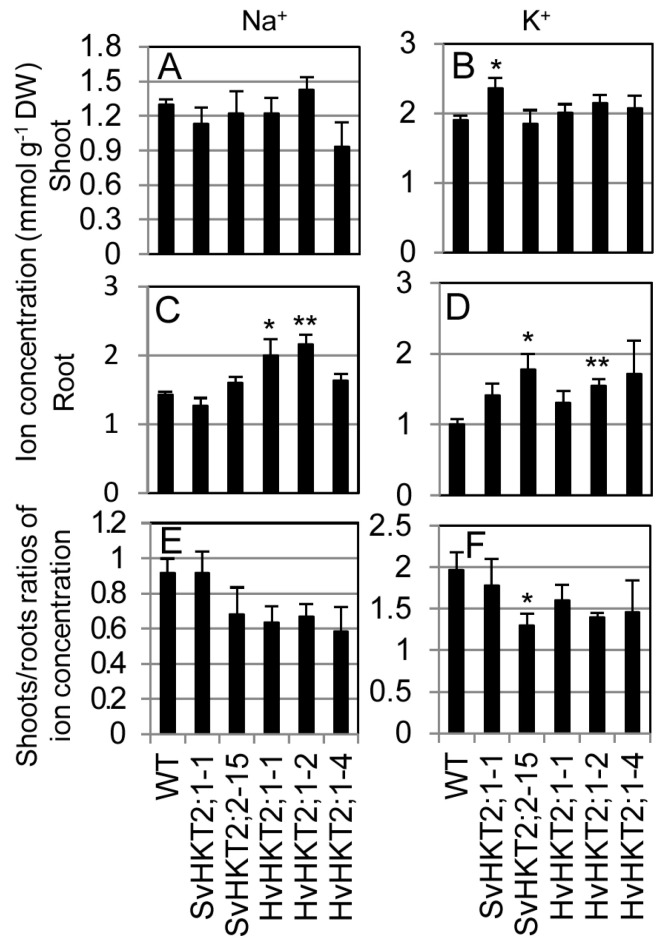
Na^+^ and K^+^ concentrations and shoots/roots ratios of Na^+^ and K^+^ concentrations in *SvHKT2s* and *HvHKT2;1* transgenic lines and WT plants grown under salt stress. (**A**–**D**) Two-week-old seedlings germinated on 1/2 MS agar medium were hydroponically cultured in 1/2 MS medium for an additional two weeks, then treated with 100 mM NaCl for another two days. Na^+^ and K^+^ concentrations in the shoots (A,**B**) and roots (**C**,**D**) and shoots/roots ratios of Na^+^ (**E**) and K^+^ (**F**) concentrations were determined. Data are presented as means ± SE (n = 3–4, biological replicates). Single and double asterisks denote significant differences compared with the values of WT plants at *p* < 0.05 and *p* < 0.01, respectively, determined using the Student’s t-test.

To determine ionic concentrations in the transgenic lines subjected to salt stress, hydroponically cultivated 3-week-old transgenic lines were subjected to 100 mM NaCl. As these lines were severely damaged after a week of this treatment, we harvested roots and shoots samples two days after the treatment and determined their respective Na^+^ and K^+^ concentrations (Figure 4). Shoot ionic concentrations were not significantly different between the transgenic lines and WT plants, except for K^+^ concentration in the *SvHKT2;1-1* line, which was significantly higher than in WT plants (Figure 4A,B). Root Na^+^ concentrations in *SvHKT2s* lines were similar to those in WT plants, while root Na^+^ concentrations in *HvHKT2;1* lines and root K^+^ concentrations in the *HKT2* transgenic lines were significantly or tend to be higher than in WT plants (Figure 4C,D). Therefore, the ectopic expression of class II HKT potassium transporters in plants could increase the accumulation of Na^+^ and/or K^+^ in roots when these were subjected to salt stress. As a result, the shoot/root ratios of Na^+^ and K^+^ tended to be lower in the transgenic lines than in WT (Figure 4E,F).

### 2.4. K^+^ and Na^+^ Concentrations in Xylem and Phloem Sap of Transgenic Arabidopsis

**Figure 5:**
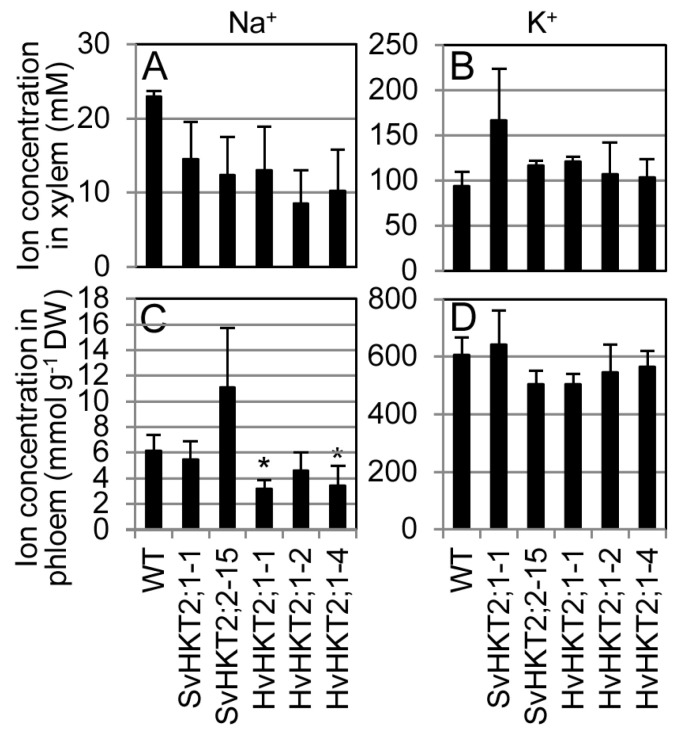
Na^+^ and K^+^ concentrations in the xylem and phloem saps of *SvHKT2s* and *HvHKT2;1* transgenic lines and WT grown on the 1/2 MS medium. Two-week-old seedlings germinated on 1/2 MS medium were hydroponically cultured in 1/2 MS medium until the bolting stage and Na^+^ and K^+^ concentrations in their xylem and phloem saps were determined. (**A**) Na^+^ concentrations in xylem, (**B**) K^+^ concentrations in xylem, (**C**) Na^+^ concentrations in phloem, and (**D**) K^+^ concentrations in phloem. Data are presented as means ± SE (n = 3-4, biological replicates). Single and double asterisks denote significant differences compared with the values of WT plants at *p* < 0.05 and *p* < 0.01, respectively, determined using the Student’s *t*-test.

To examine the effects of *HKT2*-expression on K^+^ and Na^+^ transport in *Arabidopsis* plants, *SvHKT2s* and *HvHKT2;1* transgenic lines and WT were hydroponically cultured in 1/2 MS medium and concentrations of K^+^ and Na^+^ in the xylem, and phloem saps were measured at the bolting stage (Figure 5). Our data show that Na^+^ and K^+^ concentrations in the transformants’ xylem sap were not significantly different from those from WT; however, they tended to be lower and higher, respectively, than those in WT (Figure 5A,B). The concentration of Na^+^ in the phloem sap of HvHKT2;1-1 and -4 lines was significantly lower than in WT and HvHKT2;1-2 line showed lower (but not significantly) phloem Na^+^ concentration than WT (Figure 5C). The concentration of K^+^ in the phloem sap of each *HvHKT2;1* line and SvHKT2;2-15 line was lower than in WT (Figure 5D). These results suggested that HKT2s could play a role Na^+^ and K^+^ transport depending on the ionic conditions in xylem and phloem parenchyma cells.

### 2.5. Ionic Influx and Efflux in Transgenic Arabidopsis Roots

**Figure 6:**
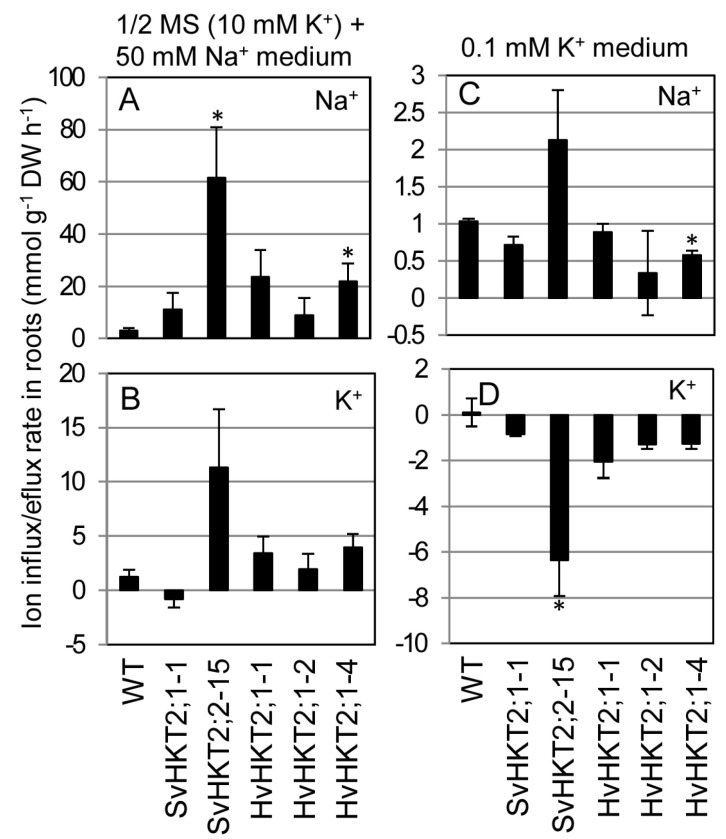
Na^+^ and K^+^ influx and efflux rate in roots of *SvHKT2s* and *HvHKT2;1* transgenic lines and WT under salinity or K^+^-starved condition. (**A**–**D**) Twelve-day-old Arabidopsis seedlings germinated on 1/2 MS agar medium were transferred into micro cuvettes filled with 50 mM Na^+^ (**A**,**B**) or 0.1 mM K^+^ (**C**,**D**) medium immersing their roots in the medium and incubated for three hours. Changes in Na^+^ (**A**,**C**) and K^+^ (**B**,**D**) concentrations in the microcuvette solution were measured, while Na^+^ and K^+^ influx and efflux rates were calculated and expressed as µM per gram of dry weight per hour (μmol g DW^–1^ h^–1^). Positive values indicate ion influx and negative values indicate ion efflux. Ten seedlings were pooled for each line and used as one sample. Data are presented as means ± SE (n = 3, biological replicates). Single and double asterisks denote significant differences compared with the values of WT plants at *p* < 0.05 and *p* < 0.01, respectively, determined using the Student’s *t*-test.

As HKT2 transgenic plants are sensitive to salt stress, it was impossible to measure ionic accumulation under saline condition. Therefore, we measured the ionic influx and efflux rates in the roots of WT and transgenic Arabidopsis seedlings to reveal the influence of SvHKT2s and HvHKT2;1 expression on ion uptake and exclusion in root epidermal cells (Figure 6). When seedlings grown in 1/2 MS medium were transferred onto the 50 mM NaCl medium, Na^+^ root influx was observed in WT and all transgenic lines, yet the influx rates were significantly higher or tended to be higher in each transgenic line compared to WT, showing that the ectopic expression of HKT2 transporters increased Na^+^ uptake in roots (Figure 6A). Similar trends for K^+^ influx were observed in roots of WT and transgenic lines, except for SvHKT2;1-1 line, which showed K^+^ efflux after 50 mM NaCl treatment (Figure 6B). In the 0.1 mM K^+^ (K^+^-starved) medium, similar levels of Na^+^ influx rates were observed in WT and all transgenic lines (Figure 6C). On the other hand, weak K^+^ efflux activities were observed in all transgenic lines, but not in WT (Figure 6D). Furthermore, the SvHKT2;2-15 line showed a significantly higher K^+^ efflux rate than WT.

## 3. Discussion

SvHKT2s (SvHKT2;1 and SvHKT2;2) and HvHKT2;1 belong to class II HKT transporter groups, as their amino acid sequence contains a glycine in the first P-loop [13,25]. Previous studies revealed that both SvHKT2s and HvHKT2;1 similarly complemented K^+^-transporter deficient yeast and mediated Na^+^ and K^+^ transport in *X. laevis* oocytes [13,25]. On the other hand, relatively little is known about the in planta functions of the class II HKT transporters. In this study, we performed a comparative analysis of the properties of these HKT2 transporters in transgenic Arabidopsis.

*HvHKT2;1* lines, as well as *SvHKT2;1* and *SvHKT2;2* lines, showed faster root growth and higher shoot Na^+^ and K^+^ concentrations than in WT under K^+^-starved conditions, despite low root ionic concentration (Figure 1 and Figure 2). This result suggested that enhanced Na^+^ and K^+^ root-to-shoot transportation and the consequent root growth promotion under K^+^-starved conditions could be characteristics common to plants overexpressing HKT2 transporters.

The mode of ionic accumulation and transport in *HKT2* expressing transgenic Arabidopsis is summarized in Table 1. Regarding K^+^ transportation, the constitutive expression of each *HKT2* tended to increase shoot K^+^ concentrations under normal conditions (non-salt stress) and K^+^-starved conditions (Figure 2G,H). Under the salt stress condition, only the *SvHKT2;1* line maintained a high shoot K^+^ concentration (Figure 4). Xylem K^+^ concentrations in the *HKT2* lines were higher (albeit not significantly and under non-stress condition) than those in WT (Figure 5B). These results suggest that HKT2s mediate K^+^ transport from the roots to the shoots by uploading K^+^ into xylem and that SvHKT2;1 plays a distinct role in K^+^ transport in salt stress conditions. In a previous study, SvHKT2;1 had higher K^+^ selectivity than SvHKT2;2 in *X. laevis* oocytes [25]. Predominant shoot K^+^ increase in *SvHKT2;1* lines subjected to salt stress may reflect this ion selectivity. On the other hand, root K^+^ concentrations decreased significantly in both *SvHKT2s* and *HvHKT2;1* lines under the K^+^-starved condition (Figure 2I). Considering these results together with the fact that K^+^ concentration in the xylem sap of transgenic lines was higher than that in WT under non-stressed conditions (Figure 5B), it was suggested that K^+^ root-to-shoot transport was enhanced by the HKT2s in the transgenic lines. Increased K^+^ uptake in the root epidermis could be also enhance K^+^ root-to-shoot transport (Figure 6B) but this is not true for the *SvHKT2;1-1* line. On the other hand, we observed that Na^+^ and K^+^ concentration in the phloem sap of transgenic lines tended to be lower than those in WT under K^+^-starved condition except for SvHKT2;2-15 line (Figure 5C,D) and that Na^+^ and K^+^ uptake rates in roots were not significantly different among the transgenic lines and WT plants under K^+^-starved conditions (Figure 6C,D). These results suggest that Na^+^ shoot-to-root translocation was repressed or Na^+^ unloading at shoot phloem parenchyma cells was enhanced in the *HKT2* transgenic lines, which resulted in Na^+^ accumulation in shoots. This hypothesis should be verified in future experiments. Furthermore, after 50 mM NaCl treatment, K^+^ influx was observed at roots of WT plants and *HvHKT2;1* and *SvHKT2;2* lines while K^+^ efflux was at *SvHKT2;1-1* line (Figure 6B), indicating a distinct function of SvHKT2;1 under salt stress. In salt tolerant rice, Pokkali, eight *OsHKT1;1* variants (V1-V8) were identified in addition to the full-length *OsHKT1;1* (FL) cDNA and OsHKT1;1-FL and -V6 exhibited inward Na^+^ rectification; however, the rests mediated bidirectional currents in both Na^+^ and K^+^ bath solutions [31]. Because in the nucleotide sequence of *SvHKT2;1,* 63 nucleotides from the 5′-end of the *SvHKT2;2* sequence were deleted, in addition to the substitution of six nucleotides [25], it is possible that *SvHKT2s* may play different roles in ion transport under specific conditions as well as the transcriptional variants of *OsHKT1;1*.

Regarding Na^+^ transportation, increased shoot Na^+^ accumulation in the *SvHKT2;1* line was observed under K^+^-starved concentrations (Figure 2G). By contrast, increased shoot K^+^ accumulation was observed in the *SvHKT2;2* and *HvHKT2;1* lines in this study (Figure 2H) and *HvHKT2;1*-overexpressing barley lines grown under K^+^-starved conditions in previous study [13]. Under salt stress conditions, only *HvHKT2;1* lines accumulated significantly higher Na^+^ concentrations in the roots compared with WT (Figure 4C). Thus, SvHKT2;2 and HvHKT2;1 could play a role in Na^+^ accumulation, while SvHKT2;1 could play a role in K^+^ transport. Arabidopsis overexpressing *PutHKT2;1* from salt-tolerant *P. tenuiflora* also had a significantly higher shoot and root Na^+^ accumulation at higher external Na^+^ concentrations or at low external K^+^ concentrations [20]. Thus, variations in the ionic transport properties of HKT2 transporters could be revealed by analysis *in planta*, that were not detected via analysis using yeast and *X. laevis* oocytes.

Transgenic barley constitutively expressing *HvHKT2;1* showed increased salt tolerance together with higher xylem and leaf Na^+^ concentration [13]. However, in this study, the overexpression of *HvHKT2;1* or *SvHKT2s* in Arabidopsis caused lower salt tolerance (Figure 2) and decreased Na^+^ concentrations in the xylem sap (Figure 5A). The overexpression of *PutHKT2;1* or *OsHKT2;1* in Arabidopsis also showed higher sensitivities to Na^+^ concentration [20]. One reason for the above differences in salt tolerance may depend on the ability to accumulate and sequestrate Na^+^ in the plants. It was reported that the plant’s ability to exclude Na^+^ is positively correlated with salinity tolerance in glycophytes, such as wheat, sorghum, maize, and tomato [32,33,34,35,36,37]; however, excluding Na^+^ from the xylem sap may not always be a plausible mechanism [38], as Na^+^ can be used as a cheap osmolyte in plants without any detrimental effects to cell metabolism by sequestrating it into the vacuole and apoplast (tissue tolerance mechanisms) [39]. Salinity treatment caused an increase in the size of palisade parenchyma cells, and this increase was much higher in the tolerant barley cultivars [38]; a similar phenomenon is common in halophytes [40]. These findings could explain why elevated shoot Na^+^ accumulation in *HvHKT1;2*-expressing barley (which already possesses high tissue tolerance mechanisms) increased salt tolerance; while in transgenic Arabidopsis (which does not possess such mechanisms), this led to increased salt sensitivity (i.e., lower salt tolerance). These results suggest that the coordinated enhancement of Na^+^ accumulation and sequestration mechanisms in shoots could be a promising strategy for conferring salt tolerance to glycophytes.

## 4. Materials and Methods

### 4.1. Transgenic Arabidopsis Plants

The production of transgenic Arabidopsis lines (SvHKT2;1-1 and SvHKT2;2-15) expressing genes *35S-SvHKT2;1* and *35S-SvHKT2;2*, respectively, was conducted according to the method described in our previous report [25]. In this study, cDNA for barley *HvHKT2;1* (AEM55590.1) was amplified by PCR using primers HvHKT1F (5′-CACCATGGGTTGGGTGAAAAGATTTTACC-3′) and HvHKT2R (5′-GTATCATACTTTCCAGGATTTACC-3′), then cloned into a pENTR vector (Thermo Fisher Scientific, Tokyo, Japan) to produce the entry vector pENTR-HvHKT2;1. The entry vector was reacted with an LR enzyme (Thermo Fisher Scientific) with a destination vector pGH1 [25] to form pGH1-HvHKT2;1, in which the transgene was driven by a CaMV35S promoter. The transformation of *Arabidopsis* WT plants (ecotype Columbia) was performed by floral dipping [41] using *Agrobacterium* strain GV3101.

### 4.2. Plant Growth Conditions and Salt Stress Treatment

Arabidopsis seeds were sown on 1/2 MS medium supplemented with 1% sucrose at pH 5.7. Plants were grown at 23 °C using a 16/8 h light/dark cycle with 60 µmol m^−2^ s^−1^ light intensity. To measure root elongation, the seedlings of transgenic and WT plants grown for 5 days on 0.1 mM K^+^ medium [25] were transplanted onto vertically positioned agar plates containing 0.1 mM K^+^ medium and incubated for an additional 5 days; finally, the root growth over 5 days was determined. To evaluate salt tolerance at the seedling stage, 7-day-old seedlings were transplanted onto 1/2 MS medium supplemented with 1% sucrose and different treatment concentrations of NaCl at pH 5.7 and plants were harvested to measure the fresh weight after another 14 days of cultivation.

*Arabidopsis* plants were hydroponically cultured using a hydroponic culture system the Home Hyponica Karen (Kyowa Co., LTD., Osaka, Japan) using 1/2 MS medium or 0.1 mM K^+^ medium supplemented with 0.2% MES at pH 5.7 as the culture solution. Fourteen-day-old plants grown on 1/2 MS agar medium were transplanted onto the hydroponic system. Plants were harvested to measure ionic concentrations and fresh weights after another 14 days of cultivation. For the salt stress treatment, 50 mM NaCl was added to 1/2 MS medium a week after transplanting onto the hydroponic system; plants were harvested to measure ionic concentrations and fresh weights after another 7 days of cultivation.

### 4.3. Real-Time qRT-PCR

Seeds of WT and transgenic seedlings were sown on 0.8% agar medium supplemented with 1/2 MS salt and 1% sucrose. Fourteen-day-old seedlings were then harvested for RNA isolation to determine the transcription levels of transgenes in transgenic Arabidopsis lines (n = 3, biological replicates). The RNA iso solution (Takarabio, Ohtsu, Japan) was used to extract total RNA and real-time qRT-PCR was performed following a previously described method [25]. To quantify the transcript of *SvHKT2;1* and *SvHKT2;2*, we used a primer pair: qSvHKT2;x-F (5′-CTTCGTCATCGTGATCTGCATCAC-3′) and qSvHKT2;x-R (5′-AGATCCTGCAGCCTCGAACAGCTG-3′) which are common to both *SvHKT2;1* and *SvHKT2;2* nucleotide sequences. For *HvHKT2;1*, we used a primer pair: qHvHKT2F (5′-CTTTGTAATAGTTGCCTGCATCACTG-3′) and qHvHKT2R (5′-AGCTGATGCAGCCTAGAACAACCG-3′), corresponding to the primer positions in *SvHKT2* nucleotide sequences. The relative expression levels of the target to the reference gene, ubiquitin extension protein (*UBQ5*, AT3G62250.1) for Arabidopsis, were calculated using the delta-delta Ct method.

### 4.4. Quantification of Ionic Concentrations in Plants

Measurement of ion concentrations in plants was performed using an Ion Analyzer IA-300 (TOA DKK, Tokyo, Japan), following the methods described in a previous report [25].

### 4.5. Collection of Xylem and Phloem Sap

Two-week-old transgenic and WT *Arabidopsis* plants cultivated on 1/2 MS plate medium supplemented with 1% sucrose at pH 5.7 for two weeks were transplanted onto liquid 1/2 MS medium supplemented with 0.2% MES at pH 5.7 until the bolting stage was reached. The collection of xylem and phloem sap was carried out according to previously reported methods [19].

### 4.6. Ionic Influx and Efflux in Transgenic Arabidopsis Roots

To measure the amount of Na^+^ and K^+^ absorbed and released by the roots, the rate of ionic influx/efflux was measured. *Arabidopsis* seeds were germinated on 1/2 MS agar medium supplemented with 1% sucrose for 12 days. Ten seedlings were then pooled for each transgenic line or WT plant and used as one sample. The roots of pooled seedlings were briefly washed in liquid 1/2 MS medium supplemented with 50 mM NaCl (50 mM Na^+^ medium) or 0.1 mM K^+^ medium, then transferred into micro cuvettes filled with 3.0 mL of 50 mM Na^+^ or 0.1 mM K^+^ medium, taking care to fully immerse the roots into the medium, and finally incubated at 23 °C under approximately 60 µmoL m^−2^ s^−1^ light intensity. Three hours after transplantation, the changes in Na^+^ and K^+^ concentrations in the microcuvette solution were measured using an Ion Analyzer IA-300 and Na^+^ and K^+^ influx/efflux rates were calculated and expressed as mM per gram of dry weight per hour (mM gDW^–1^ h^–1^).

## Figures and Tables

**Table 1:** Mode of ionic accumulation in *HKT2*-expressing plants in comparison to the WT.

Plant Part	1/2 MS Medium		Low K^+^ Medium		100 or 50 mM NaCl Medium		Data Source
	Na^+^	K^+^	Na^+^	K^+^	Na^+^	K^+^	
Shoots Roots	↓ ↑ ↑↑	↑ –	↑↑ (SvHKT2s) ↑ (HvHKT2;1) ↓↓	↑↑ ↓	– – (SvHKT2s) ↑↑ (HvHKT2;1)	↑↑(SvHKT2;1) – (Others) ↑, ↑↑	Figure 2 Figure 4
Xylem Phloem	↓ – (SvHKT2s) ↓↓(HvHKT2;1)	↑ ↓	ND ND	ND ND	ND ND	ND ND	Figure 5
Roots	ND	ND	–	↓↓(SvHKT2;2) ↓(Others)	↑, ↑↑	↓ (SvHKT2;1) ↑ (Others)	Figure 6

Note: ↑, ↑↑, ↓, ↓↓, and – indicate increasing tendency, a significant increase, decreasing tendency, significant decrease, and no change in ionic accumulation, respectively, in the indicated organ/tissue of *HKT2* transgenic lines compared to those of WT plants. ND: not determined.
